# Development of an Active Optical Lens for Arc Flashing Detection

**DOI:** 10.3390/s23052629

**Published:** 2023-02-27

**Authors:** Paweł Awramiuk, Karolina Sadowska, Jarosław Wiater, Dariusz Sajewicz, Marcin Kochanowicz, Wojciech Walendziuk, Jacek M. Żmojda

**Affiliations:** Faculty of Electrical Engineering, Bialystok University of Technology, Wiejska 45D, 15-351 Bialystok, Poland

**Keywords:** optical lens, PMMA, lanthanide ions, UV sensor

## Abstract

This paper contains the design of active optical lenses used for the detection of arc flashing emissions. The phenomenon of an arc flashing emission and its characteristics were contemplated. Methods of preventing these emissions in electric power systems were discussed as well. The article also includes a comparison of commercially available detectors. An analysis of the material properties of fluorescent optical fiber UV-VIS-detecting sensors constitutes a major part of the paper. The main purpose of the work was to make an active lens using photoluminescent materials, which can convert ultraviolet radiation into visible light. As part of the work, active lenses with materials such as Poly(methyl 2-methylpropenoate) (PMMA) and phosphate glass doped with lanthanides, such as terbium (Tb^3+^) and europium (Eu^3+^) ions, were analyzed. These lenses were used to make optical sensors, which were supported by commercially available sensors in their construction.

## 1. Introduction

Today, new energetic systems use innovative devices that enhance reliability and working safety. Such systems are usually complex and expensive; therefore, they must have equally modern and indefectible anti-short-circuit protection devices. A burning electric arc generates a high financial cost, and for this reason, new systems based on various ways of quickly detecting and preventing short circuits are still required. One method of fast arc flash detection in anti-short-circuit protection is applying optical fiber sensors made of PMMA [1]. Despite the high efficiency in detecting burning arcs by the fiber sensors, the operating of the anti-short circuit causes a flash of several thousand luces. For this reason, the use of lens point sensors allows better control in small confined spaces. Proper installation of the sensors and relays provides logical detection and trip points in any system. It should also be remembered that electrical discharges are lethal to humans. Therefore, a burning electric arc must be extinguished very quickly. Such a solution should increase the level of safety of people working in the vicinity of machines and elements under electrical voltage [2]. Having in mind the physics of the phenomenon of forming an electric arc, which appears shortly before the flash partial discharge, we propose a modification of the optical fiber sensor based on luminescent materials. Partial discharges are accompanied by electromagnetic waves, which also cover UV light radiation. Depending on the medium in which the discharge occurs and the atmospheric factors, the emission spectrum is usually within the ultraviolet radiation range [3]. Therefore, it seems crucial to apply an optical fiber sensor which can convert the UV radiation from a partial discharge to an emission within the visible range. The detection of UV radiation may be used to detect a forming electric arc before the destructive flash. Such a solution increases the probability of the arc short-circuit’s elimination, which means that it protects the switchgear from damage caused by a burning electric arc.

One group of materials which can be used for constructing such sensors are polymers, i.e., multimolecular chemical compounds. Methyl polymethacrylate (PMMA) is a material with effective transmission properties and it is easy to mechanically process. It is also robust to the influence of water, petrol, acids, and weather conditions (including low temperatures) [3,4].

The most effective luminescent processes occur when xanthenic, anionic, and aromatic hydrocarbon dyes are used [5,6,7,8,9,10,11,12]. They are characterized by a wide excitation band and a short luminescence decay. Industrial dyes, such as perylene or lumogen, are also efficient dopants to transparent polymers because of their fluorescent properties. These materials are characterized by strong luminescence under ultraviolet radiation excitation [13,14,15,16,17]. Moreover, Miluski et al. showed that PMMA doped with 1,4-Bis (2-methylstyryl)benzene (BIS-MSB), which has a high quantum efficiency, could intensely absorb UV radiation, and it emitted luminescence at the maximum wavelength of 450 nm [18].

Another alternative group of materials is glasses doped with rare earth elements (REE). As a result of doping with trivalent ions of the lanthanide elements, the glasses obtain some specific properties depending on the electron structure. The structure determines the characteristic wavelengths at which the selective absorption and emission bands can occur [19,20,21,22]. Among all lanthanides, Eu^3+^ and Tb^3+^ ions are good candidates for UV detection, due to their narrow absorption bands and high quantum efficiency [23,24,25,26]. Additionally, the 5d→4f transition allows a wide UV absorption band, which effectively enhances the luminescence band at the wavelength of 540 nm for Tb^3+^ ions and at the wavelength of 620 nm for the Eu^3+^ ions. Other important features of optical glasses, which should be discussed, are the UV spectral window and the transmission level. In the case of applying them in UV sensors, phosphate glasses are characterized by a 97% level of spectral transmission and a wide optical window in the range from 250 nm to 2000 nm. Moreover, the high thermal stability of phosphate glasses allows them to form without a crystallization effect at a high concentration of the rare earth elements in the matrix (up to 7% of mass), which significantly increases the sensitivity [27,28,29,30].

In the literature, many optical fiber luminescent sensors constructed with the use of PMMA technology have been described. We found descriptions of relatively simple solutions, which enable detection from the head of the fiber equipped with an optically active element, as well as more complex ones, which are based on the detection carried out by the side face of the fiber [31,32,33,34,35]. Lyons et al. applied in their research the photoluminescence phenomenon of a specially prepared active layer, absorbing UV radiation, spread on the core of a polycarbonate optical fiber. This solution enabled multipoint monitoring of the UV radiation that fell on the surface of three different luminophores (red, green, and blue) [36]. Another interesting sensor construction was the photothermal-refractive glass fibers with silver and semiconductor molecular clusters, which have optimized parameters for UV radiation detection from the point of view of the spectral region of UV excitation and visible luminescence [37].

In this paper, the results of the experimental investigations on the analysis of the luminescent properties to confirm the detection abilities of the developed active lenses are presented. The constructed sensors are compared with the commercially available measuring heads.

## 2. Materials and Methods

Having in mind the possibility of converting the UV radiation in various optical fiber constructions of the luminescence sensors, we focused on the possibility of applying the polymer materials and optical glasses used for constructing active lenses compatible with real systems of anti-short-circuit protection. Within the research, active lenses were made of polymer materials (PMMA) doped with dyes, as well as of phosphate glass doped with europium and terbium ions.

### 2.1. PMMA Sensor

**Figure 1:**
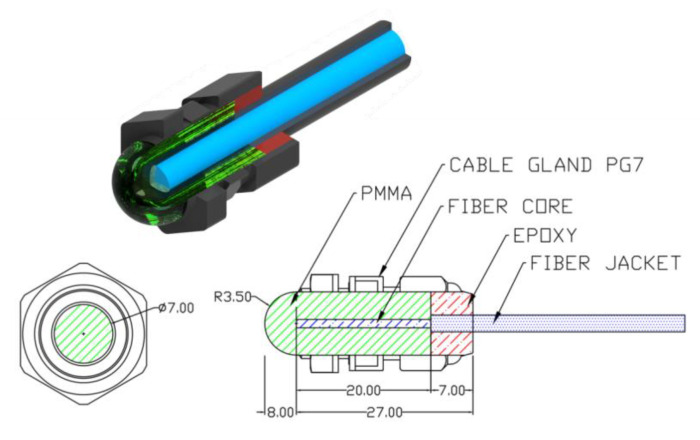
Cross sections of the mechanical structure of the active optical lens made of PMMA, poly(methyl methacrylate).

**Figure 2:**
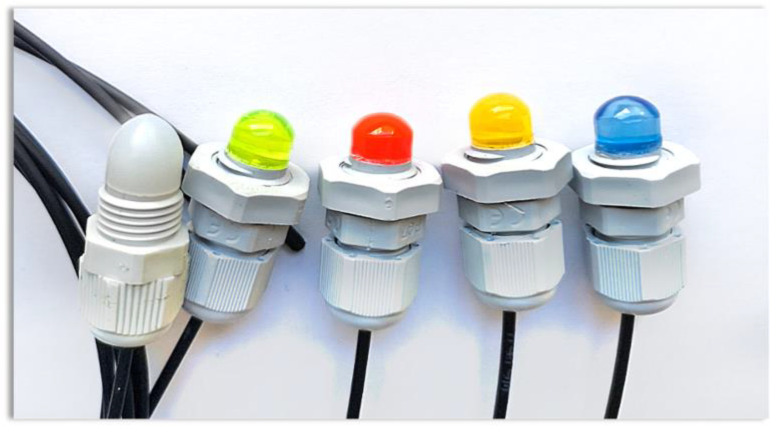
Series of fabricated luminescent sensor heads.

A design of a measuring head exploiting an active polymer lens with the possibility of detecting UV radiation is presented in Figure 1. In order to produce the active polymer lenses, the PMMA doped with red, yellow, green, and blue dyes and 1,4-Bis(2-methylstyryl)benzene (BIS-MSB) were used. Rods of 7 mm diameter were processed mechanically by grinding and polishing to give them the target shape of lenses. The coupling of the active sensor with the transition optical fiber was executed by placing a 20 mm fragment of the transition optical fiber in a drilled slot (Ø = 2 mm). The whole construction was placed in a plastic cable gland and deluged with epoxy resin to strengthen the connection and its endurance (Figure 2).

### 2.2. Glass-Based Sensor

**Figure 3:**
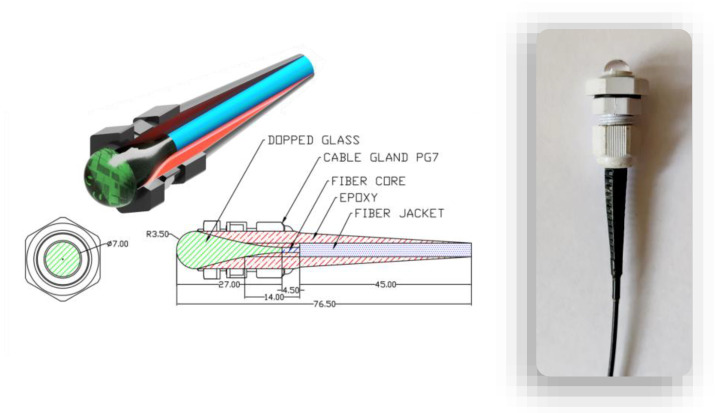
Glass-based sensor head cross sections and the tested sensor’s general view.

Figure 3 presents a design of the glass-based sensor. In order to produce the luminescent glass lenses, rods were made of phosphate glasses and doped with 2 mol.% of tEu_2_O_3_ and Tb_2_O_3_). The chemical composition of the glasses was as follows: 65P_2_O_5_–9Al_2_O_3_–7BaO–6ZnO–5Na_2_O–6MgO–2Eu_2_O_3_/2Tb_2_O_3_ (Table 1 and Table 2) [38]. Then, 15-gram samples were melted in quartz crucibles placed in a furnace heated up to 1350 °C. The prepared liquid substance was poured into a brass cylindrical form. Then, the obtained glass was annealed in a furnace at 450 °C for 8 h to minimize the mechanical strains. Out of the 15-gram sample, rods of a diameter of 7 mm and a length of 60 mm were formed. They were later placed in a tube furnace of an optical fiber drawing tower.

The fabrication process was as follows. Firstly, the glass rod was placed in a tube furnace at the temperature of 830 °C. Next, the glass rod was moved vertically in the furnace heating zone, which caused the softening of the glass. Through the gravity force and appropriate heating process, an optical sensor head with a droplet shape was formed (Figure 3). In the final stage of the procedure, the material was trimmed, and the surface connected with the optical fiber core was polished. In addition, in this case, the lens was placed in a plastic cable gland and strengthened with the use of epoxy resin.

### 2.3. Laboratory Test Bench for the Optical Characterization of the Sensor Heafds

**Figure 4:**
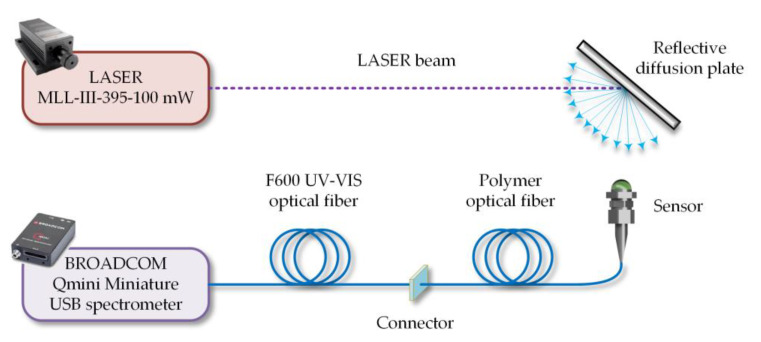
Experimental setup of the luminescent measurements of the fabricated sensor heads.

**Figure 5:**
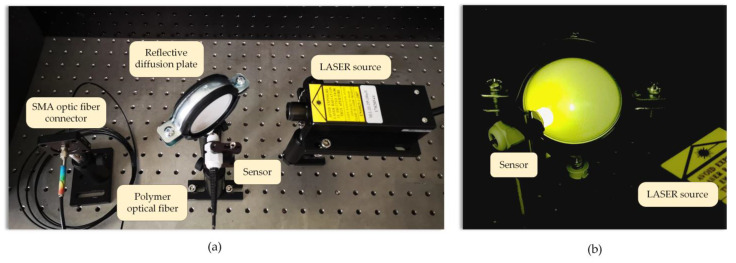
General view of the laboratory test stand (**a**) and experiment in which the illumination of the sensor head under UV laser excitation is shown (**b**).

The transmission spectra within the range from 300 nm to 800 nm were measured in a measurement chamber equipped with a miniature Deuterium and halogen light source by StellarNet and a spectrometer by Broadcom Qmini with a Silicon (Si) detector. The spectra of luminescence were measured with the use of the system presented in Figure 4 and Figure 5. The designed and built laboratory stand was composed of a 395 nm semiconductor laser, a diffuse plate, and a fabricated sensor, which was placed on the handle and connected to the spectrometer with the use of an SMA connector. The diffuse plate enabled laser beam diffusion and a homogenous distribution of the radiation density, which helped to achieve conditions similar to the radiation emission when a real arc is formed.

**Figure 6:**
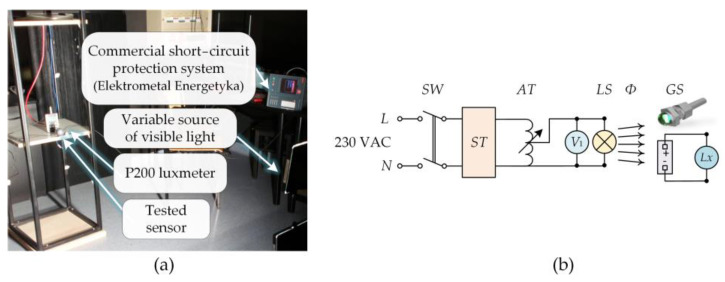
Laboratory test bench (**a**) and the electrical diagram of the measuring system (**b**) for analysis of the activation threshold of the developed heads. The electrical circuit (**b**) contained: *ST*—voltage stabilizer, *AT*—autotransformer, *LS*—light source, *V*_1_—digital voltmeter, *Φ*—luminous flux, *Lx*–lux meter.

The tests of the threshold of the developed heads in the condition of daylight were conducted in a darkroom with the use of radiators equipped with halogen light sources. A diagram of the measurement system for measuring light intensity is presented in Figure 6. The tests were conducted in conditions similar to the real ones (voltage switchgear). The specially produced sensors were connected to a commercial anti-short-circuit protection system manufactured by Electrometal Energetyka, e^2^TANGO 800 Bay Controller [39]; thus, it was possible to obtain the information concerning the value of the light intensity *(Lx)* received by the sensor, which would cause the disconnection of the power circuit. The change in the light intensity of the halogen light source *(LS)* received by the head *(GS)* was performed by changing the voltage supply level (*V*_1_) on the autotransformer clamps *(AT)*. The value of the radiation intensity was recorded by a Sonopan P200 lux meter [40] whose head was placed within the plane of the tested optical fiber sensor.

## 3. Results and Discussion

The analysis of the spectroscopic properties of the selected materials was conducted to confirm their ability to detect ultraviolet radiation.

**Figure 7:**
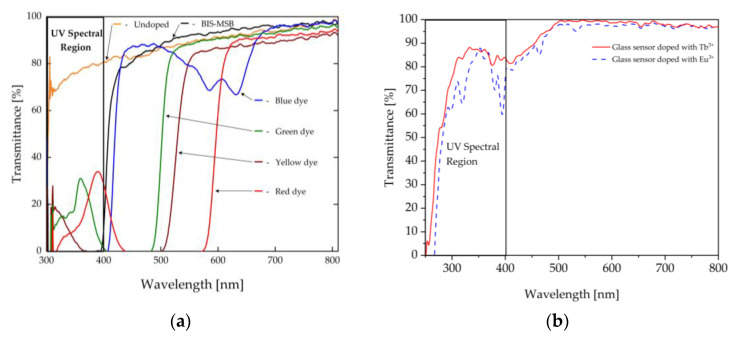
Transmission spectra of (**a**) the PMMA materials doped with dyes and (**b**) the phosphate glasses doped with Eu^3+^ and Tb^3+^ ions used in the sensor construction.

Figure 7a,b present the transmission spectra of the polymer and glassy materials used to construct the active lenses. As can be observed, a nondoped polymer (orange color) had the lowest level of UV transmission, which significantly limited its use as an effective source of UV propagation. For this reason, PMMA is exploited in commercial sensors only as a transmission medium of a burning arc (visible spectrum). Based on the conducted tests, the absorption edge of the doped PMMA shifted toward the longer wavelengths of the radiation, in compliance with the applied dye. Two polymers seemed to be of special interest. One of them was the blue color polymer, which had a wide absorption band within the range of 300 nm to 400 nm and additional bands at wavelengths of 580 nm and 620 nm. The other one was the BIS-MBS polymer, which had a strong band within the range of 300 nm to 400 nm, i.e., the analyzed range of the arc emission in a partial discharge. The remaining dyes had a lower level of absorption within the tested range.

In the same way, measurements of the spectral transmission of the developed phosphate glasses doped with europium and terbium ions were conducted (Figure 7b). Those ions had relatively weak and narrow absorption bands within the range of 300 nm to 400 nm, in which the Eu^3+^ ions had a strong absorption band at the wavelength of 395 nm, which corresponded to the ^7^F_0_→^5^L_6_ transition. The Tb^3+^ ions had an absorption band at the wavelength of 378 nm (^7^F_6_→^5^D_3_ transition) [41].

**Figure 8:**
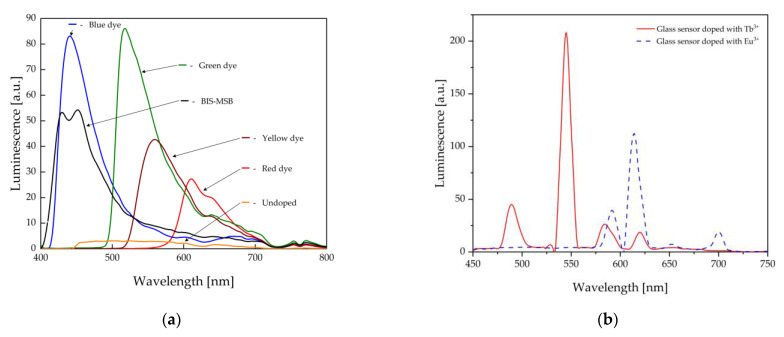
Luminescence spectra of (**a**) the PMMA and (**b**) the phosphate glass of the fabricated sensor heads.

In order to confirm the electrical arc detection ability of the produced heads, measurements of the luminescence spectra within the range of 400 nm to 800 nm were conducted (Figure 8a,b). In each tested case, wide luminescence spectra resulting from the doped organic dyes were achieved. Based on the analysis of the obtained results, in the case of the PMMA sensors, the best conversion performance at the radiation of 395 nm was revealed by the sensors made of the rods doped with the following dyes: blue, green, and BIS-MSB. 

The relatively low luminescence intensity of the BIS-MSB sensor under 395 nm excitation resulted from a low level of dye in the polymer. Moreover, it was observed that in the case of the commercial PMMA sensor, under the UV exposition, the fluorescence level was scarce; thus, effective detection was impossible. In the case of the measurements conducted for the developed heads with active glass lenses (Figure 8b), strong and narrow emission bands within the range of 500 nm to 700 nm under UV exposition were observed. The spectral response of the sensor with the Eu^3+-^ doped head showed the strongest luminescence band at the wavelength of 620 nm, which was close to a red color, whereas the sensor doped with Tb^3+^ ions was distinctive at the emission band wavelength of 540 nm, which was similar to a green color. The obtained luminescence characteristics of the glasses confirm that, despite a smaller absorption within the UV radiation, they had a high emission intensity, which was easy to detect.

The tests of the functionality of the developed optically active sensors were also conducted in the conditions as close as possible to those that occur in the real anti-short-circuit protection systems. The influence of the intensity of the daylight radiation on the head, which can appear in the switchgear during servicing (when the switchgear door is opened, the circuit is disconnected), was one of the crucial parameters measured by the sensors. Table 3 shows the values of the visible radiation intensity obtained for the chosen PMMA sensors and the glass-based sensors in comparison with the values of the commercial sensors. Based on a preliminary analysis, it was observed that the detection threshold of the specially produced PMMA sensors was lower than 1000 lx, whereas in the case of glass sensors, the detection level increased up to almost 3000 lx. However, the obtained detection levels were much lower than those for the commercial solutions (10,000 lx). Thus, direct use of the sensors produced in this research as potential replacements for commercial ones seems to be impossible currently. Nevertheless, having in mind their main role, which is detecting UV radiation from a partial discharge, the possibility of using them as complementary elements in modern protection systems seems to be significant.

## 4. Conclusions

This research analyzed the potential of using luminescent materials as detectors of the UV radiation coming from a partial discharge of an electric arc. Two types of materials in the form of dye-doped polymers and phosphate glasses doped with europium and terbium ions were fabricated and analyzed. Optical fiber sensors, based on a construction of commercial sensors applied in anti-short-circuit protection systems, were constructed with the use of the selected materials. Based on the luminescence measurements, it was confirmed that all the tested materials enabled detecting the UV radiation, although each to a different extent. A systematic analysis allowed us to state that the sensors made of phosphate glass revealed a greater response signal to the UV radiation excitation, despite narrower absorption bands, in comparison with the PMMA dyes. Moreover, the worked-out glass sensor heads had a higher excitation edge at daylight radiation (>2000 lx), which compared with the PMMA sensors (>600 lx) makes them an alternative material to be applied in anti-short-circuit protection systems. 

The most important achievements of the presented research include:

- producing phosphate glasses doped with europium and terbium ions;
- conducting measurements of the luminescence spectra of the produced glasses;
- making lenses with the use of polymers and doped glasses;
- designing and practically realizing sensors with the produced glasses;
- measuring the luminescent properties and sensitivity of the produced sensors in laboratory conditions;
- conducting an analysis of the absorption properties of the selected fluorescent materials (polymers and doped glasses) consisting in determining the ability to detect radiation within the ultraviolet spectrum;
- conducting measurements of the emission bands of the polymers doped with dyes.

Having in mind the obtained results, in the next stage of the research, it is planned to conduct in situ tests, where as a result of a partial discharge appearing in the blade generator, it will be possible to detect the emission from a sensor doped with the lanthanides ions. Additionally, it is planned to construct an electronic system for the electric switchgear supply disconnector in which the worked-out sensors will be applied.

## Figures and Tables

**Table 1:** Compositions of phosphate glasses doped with europium ions (2% Eu^3+^).

Ingredients	Molar Content [mol.%]	Mass [g]
P_2_O_5_	65	10.678
Al_2_O_3_	9	1.062
BaO	7	1.242
ZnO	6	0.565
Na_2_O	5	0.358
MgO	6	0.280
Eu_2_O_3_	2	0.815
Σ	100	15.000

**Table 2:** Compositions of phosphate glasses doped with terbium ions (2% Tb^3+^).

Ingredients	Molar Content [mol.%]	Mass [g]
P_2_O_5_	65	10.655
Al_2_O_3_	9	1.060
BaO	7	1.240
ZnO	6	0.564
Na_2_O	5	0.357
MgO	6	0.279
Tb_2_O_3_	2	0.845
Σ	100	15.000

**Table 3:** The detection level of sensors by diffuse daylight.

Sensor	Detection Level [lx]	Ref.
Energotest (PMMA)	10,000	[42]
Arcteq Relays (point sensor, fiber optic loop)	8000	[43]
Yellow PMMA	678	This work
Red PMMA	622	This work
Eu^3+^ Glass	2431	This work
Tb^3+^ Glass	2788	This work

## Data Availability

Not applicable.
